# “Farmácia Dose Certa” program and Beers-Fick criteria: general *versus* specific analysis

**DOI:** 10.1590/S1516-31802012000400013

**Published:** 2012-09-04

**Authors:** Giancarlo Lucchetti, Alessandra Lamas Granero Lucchetti, Sueli Luciano Pires, Milton Luiz Gorzoni

**Affiliations:** I MD. Geriatrics Specialist, Geriatrics and Gerontology Sector, Santa Casa de Misericórdia de São Paulo, São Paulo, Brazil.; II MD. Geriatrics Specialist, Interdisciplinary Center for Aging Research and Care, Belo Horizonte, Minas Gerais, Brazil.; III MD, MSc. Professor of Geriatrics and Director of Hospital Dom Pedro II, Geriatrics and Gerontology Sector, Santa Casa de Misericórdia de São Paulo, São Paulo, Brazil.; IV MD, PhD. Professor and Head of Geriatrics and Gerontology Sector, Santa Casa de Misericórdia de São Paulo, São Paulo, Brazil. Geriatrics and Gerontology Sector, Santa Casa de Misericórdia de São Paulo, São Paulo, Brazil.

Dear Editor,

First, we would like to thank the authors of the letter: “Drugs available through the Farmácia Dose Certa program and Beers criteria: a further analysis” for their comments. Our group has been working on polypharmacy, the Beers-Fick criteria and adverse drug effects among the geriatric population for many years.^1^^,^^2^^,^^3^ In fact, our article^4^ had the main purpose of alerting health professionals who deal with older individuals to the risks involved in the drugs available through the Dose Certa program in general, and not for those with specific medical conditions.

Due to space restrictions, we decided to consider only the first Beers-Fick criterion “drugs or drug classes that should generally be avoided for people over 65 years of age, because they are ineffective or have a high risk of unnecessary adverse effects when a safer alternative is available”. The second criterion, which should be considered according to specific known conditions, would be very complex for this paper, because for each medical condition, there is a full range of drugs that must be avoided. For instance, those with stress incontinence may not use the following drugs: alpha-blockers (doxazosin, prazosin and terazosin), anticholinergics, tricyclic antidepressants (imipramine hydrochloride, doxepin hydrochloride and amitriptyline hydrochloride) and long-acting benzodiazepines.

In order to ease the interpretation of our article, we decided to include only the first Beers-Fick criterion. Nevertheless, a further article dealing with the second criterion could be also helpful.

We believe that our paper has fostered discussion about whether geriatric patients should be treated more carefully than the general population, in public healthcare programs.
